# Supplementary material for: The TLR4/NFκB-Dependent Inflammatory Response Activated by LPS Is Inhibited in Human Macrophages Pre-Exposed to Amorphous Silica Nanoparticles
Source: Nanomaterials (Basel). 2022 Jul 5;12(13):2307. doi: 10.3390/nano12132307 (PMC9268534; doi:10.3390/nano12132307)
Supplement: Supplementary file 1 [file nanomaterials-12-02307-s001.zip › nanomaterials-1757851-supplementary.pdf]

nanomaterials

Article

The TLR4/NFκB-dependent inflammatory response activated by LPS is inhibited in human macrophages pre-exposed to amorphous silica nanoparticles.

Massimiliano G. Bianchi [1,*], Martina Chiu [1], Giuseppe Taurino [1], Enrico Bergamaschi [2], Francesco Cubadda [3], Guido M. Macaluso [1,4] and Ovidio Bussolati [1,5].

Supplementary Figure

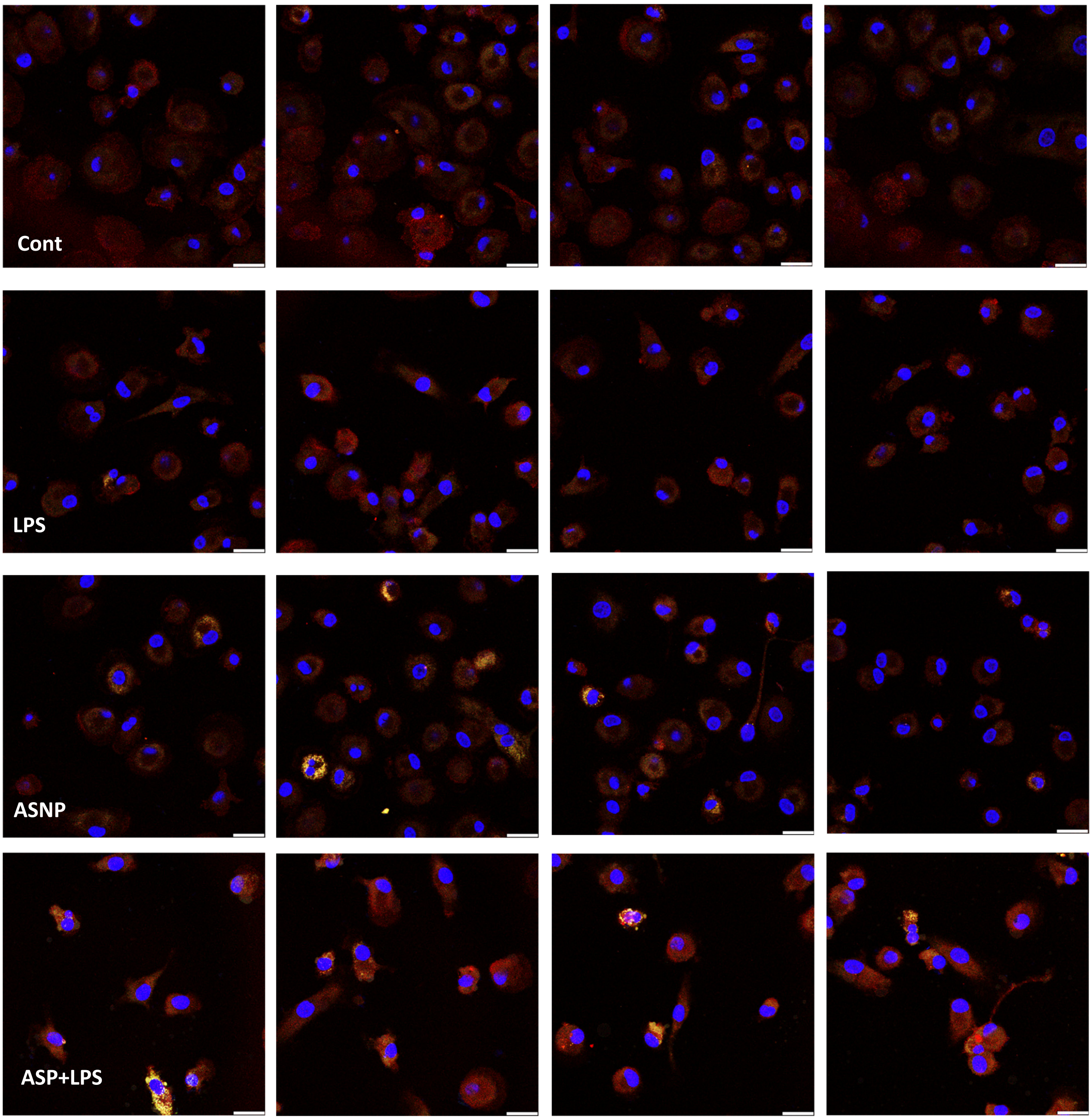

Supplementary Figure S1. ASNP effect on TLR4 distribution in human MDM. Macrophages were treated as described in figure 3. Confocal single sections of four representative fields of MDM cultures stained for TLR4 (red) and LAMP1 (green). Nuclei are counterstained in blue (Bars = 20 μm).
